# Optimization of Curvilinear Stiffener Beam Structures Simulated by Beam Finite Elements with Coupled Bending–Torsion Formulation

**DOI:** 10.3390/ma16093391

**Published:** 2023-04-26

**Authors:** Cesare Patuelli, Enrico Cestino, Giacomo Frulla, Federico Valente

**Affiliations:** 1 Department of Mechanical and Aerospace Engineering (DIMEAS), Politecnico di Torino, Corso Duca degli Abruzzi 24, 10129 Torino, Italy; cesare.patuelli@polito.it (C.P.); enrico.cestino@polito.it (E.C.); 2 ITACAe S.r.l., Via Calosso 3, 14100 Asti, Italy; f.valente@itacae.com

**Keywords:** bending–torsion coupling, curvilinear stiffeners, beam finite elements, additive manufacturing, topology optimization

## Abstract

This research presents the application of a beam finite element, specifically derived for simulating bending–torsion coupling in equivalent box-beam structures with curvilinear stiffeners. The stiffener path was simulated and optimized to obtain an expected coupling effect with respect to four typical static load cases, including geometric constraints related to the additive manufacturing production method. The selected load condition was applied to the centroid of the beam section, and the structure performance was consequently determined. A variation in load position up to one-fourth of the beam width was considered for investigating the stiffener path variation corresponding to a minimum bending–torsion coupling effect. The results demonstrated the capability of such a beam finite element to correctly represent the static behavior of beam structures with curvilinear stiffeners and show the possibility to uncouple its bending–torsion behavior using a specific stiffener orientation. The simulation of a laser powder bed fusion process showed new opportunities for the application of this technology to stiffened panel manufacturing.

## 1. Introduction

High aspect ratio wing configuration and weight reduction can improve aircraft energy efficiency, reducing CO_2_ emissions to match the standards adopted by the 36-state ICAO council. The resulting slender structures are highly flexible and are subjected to aeroelastic instabilities [1,2,3,4,5], both static and dynamic. Divergence is a typical static aeroelastic instability involving torsion deformation, which is potentially increased up to an unsafe level. Bending–torsion flutter is referred to as a classical aeroelastic dynamic instability that causes rapidly increasing amplitude oscillations up to a dangerous extent if related to the wing structural integrity. Therefore, specific design care has to be devoted to the definition of innovative structural configurations capable of mitigating such critical phenomena. Anisotropic materials can be adopted to enhance wing box structural performances with no weight penalties by combining both aerodynamic and material coupling, according to the concept of “aeroelastic tailoring”, as described in the review papers of Jutte and Stanford [6] or of Shirk et al. [7].

Aeroelastic tailoring demonstrates its important advantages when orthotropic materials are involved in the design. Composite material lay-ups can be optimized to obtain a desired behavior in connection to functionally graded materials (FGM) [8,9,10,11] and variable angle tow (VAT) [12,13,14,15,16]. An alternative solution can be based on curvilinear stiffened panels, as described in [17,18]. All these technologies can be adopted as passive aeroelastic tailoring.

The optimization of material orientation for aeroelastic tailoring has been widely investigated and enlarges the design space for next-generation wings. Weisshaar [19] used a set of closed-form solutions on static aeroelastic problems to describe the effects of fibrous composites on aerodynamic characteristics of composite wings and illustrated the ability of aeroelastic tailoring to modify the spanwise center-of-pressure location.

Kameyama et al. examined [20] the effects of laminate configurations on flutter and divergence characteristics of composite plate wings with various sweep angles and conducted an optimization to find the minimum weight design, with constraints on the flutter and divergence speeds. Stroud et al. [21] presented an approach for the reliability-based optimization of metallic wing plates to meet strength and flutter requirements. The design variable was the thickness distribution, while the constraints were the weight and the probability of failure. Maute et al. [22] presented a topology optimization methodology for the design of aeroelastic structures, accounting for the fluid–structure interaction. The optimization results showed the significant influence of the design dependency of the loads on the optimal layout of flexible structures when compared with results that assumed a constant aerodynamic load. Other works involving aeroelastic tailoring and composite materials were presented in [23,24,25,26].

The optimization process can be very demanding in terms of time and computational costs. Equivalent models or beam elements can be adopted to find optimal solutions for the early design stages. Danzi et al. [27] used an equivalent continuum plate model to obtain an optimal configuration through a topology optimization problem, where the design variables became the orientation of the stiffeners at prescribed points.

**Figure 1:**
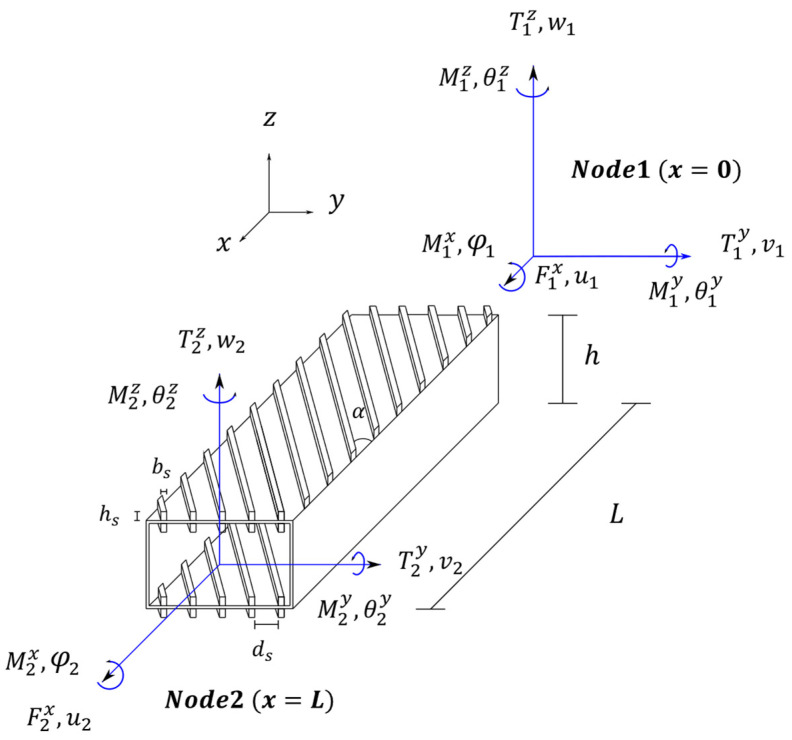
Beam element reference system with dimensions, nodal degrees of freedom, and resultants [28].

In the present work, a beam finite element with bending–torsion coupling formulation (Figure 1), developed and validated with numerical and experimental results by Patuelli et al. [28,29], was adopted to find the optimal configuration of curvilinear stiffener panels of a box-beam structure. The bending–torsion coupling beam finite element (BTCE) was used in a topological optimization to find the optimal stiffener orientation that guaranteed prescribed levels of deflection and torsion for three different load cases (LC1, LC2, and LC3) located on the beam axis; these design cases were identified as LC1_C, LC2_C, and LC3_C. A second optimization procedure was performed with the loads positioned at one-fourth of the beam width, imposing 0-tip torsion angles as constraints and thus generating uncoupled bending and torsion behavior. These optimizations were identified as LC1_U, LC2_U, and LC3_U. The analysis was limited to static load cases to reduce the number of design variables and to test the capabilities of the derived beam finite elements in the presence of curvilinear stiffeners; moreover, this work aimed to verify the existence of an optimal configuration that can couple or decouple bending and torsion within prescribed constraints.

An additional optimization procedure was considered to obtain a self-supporting structure in the additive manufacturing production scheme by selecting the right stiffener orientation for minimizing the support structure extension and weight. General guidelines are available in literature and the ISO/ASTM normative [30,31,32]. Additionally, for the definition of the best build orientation for the minimization of the support structure, other criteria were considered for the design of the product and the process, such as surface finish, powder and part removal ease, inspection accessibility, and others. The presence of support structures can increase time and costs for AM parts production; for this reason, the self-support requirement was taken as a technological constraint in this case. This optimization was identified with the acronym LC_AM.

A static analysis of the resulting optimal configurations subjected to the related loads was performed with two finite element (FE) models: a SHELL FE model and a TETRA10 FE model. The static results were compared to the ones computed by the BTCE model to verify its simulation capabilities in the presence of curvilinear stiffeners and its performance as an optimization tool. The manufacturing of a portion of the optimized beam by means of the laser powder bed fusion (LPBF) process was simulated with the software AMTOP^®^ V. 2.0, which was developed by ITACAe S.r.l. Asti, Italy and SimTech Simulation et Technologie SARL Paris, France for enhancing the AM application to the curvilinear stiffener box-beam configuration. The software was based on the FE discretization of the macro-scale process; this class of models can be used to understand the overall temperature progressions and deformations during the process, as shown in [33,34,35,36,37,38].

The objective of this work is to investigate the capabilities of the BTCE as an optimization tool to find a desired box-beam structure configuration at a very early design stage and to assess its accuracy in determining structural performances in the presence of curvilinear stiffeners. Moreover, the presented research explores the possibilities of AM production for such a stiffened configuration, taking into consideration related geometric constraints.

The research paper is organized as follows: the second section briefly presents the characteristics of the BTCE, with a focus on its derivation. In the third section, the geometry of the box-beam structure, the optimization problems, and the finite element models are described. The fourth section contains the results, while the conclusions are outlined in the fifth and last sections).

## 2. Finite Element Model

The finite element used in this work was a two-node finite element with six degrees of freedom per node, derived by Patuelli et al. [28], which included bending–torsion couplings; the elongation was not considered in this work, so the nodal degrees of freedom were reduced to five. The derivation proceeded from the equation of motion derived according to [39,40], considering only linear terms:

(1) $$\left\{\begin{array}{c} m\frac{\partial^{2}v}{\partial t^{2}}+C_{33}\frac{\partial^{2}}{\partial x^{2}}\left(\frac{\partial^{2}v}{\partial x^{2}}\right)=0\\ m\frac{\partial^{2}w}{\partial t^{2}}+C_{22}\frac{\partial^{2}}{\partial x^{2}}\left(\frac{\partial^{2}w}{\partial x^{2}}\right)-C_{12}\frac{\partial }{\partial x}\left(\frac{\partial^{2}\varphi }{\partial x^{2}}\right)=0\\ \rho I_{p}\frac{\partial^{2}\varphi }{\partial t^{2}}-C_{11}\frac{\partial }{\partial x}\left(\frac{\partial \varphi }{\partial x}\right)+C_{12}\frac{\partial }{\partial x}\left(\frac{\partial^{2}w}{\partial x^{2}}\right)=0 \end{array}\right.$$

where $C_{22}$ and $C_{33}$ are the bending stiffness with respect to the *y-* and *z*-axes, $C_{12}$ represents the bending–torsion coupling coefficient, $C_{11}$ is the torsional stiffness, m=ρA is the mass of the beam element, ρ is the material density, and $I_{p}$ is the polar moment of inertia of the beam section. The edgewise, spanwise, and torsional displacements were represented as v, w, and φ, respectively, and were defined in the spatial coordinate x and in the time t.

The structure considered in this work was a circumferentially asymmetric stiffness (CAS) configuration, and the stiffness coefficients were obtained with Equation (2).

(2a) $$C_{00}=\oint A_{11}^{*}ds,$$

(2b) $$C_{11}=\frac{4\Omega^{2}}{\oint {(1/A}_{66}^{*})ds}+4\oint D_{66}^{*}ds,$$

(2c) $$C_{12}=2\Omega \frac{\oint {(A_{16}^{*}/A}_{66}^{*})ds}{\oint {(1/A}_{66}^{*})ds}+4\oint D_{66}^{*}ds,$$

(2d) $$C_{22}=\oint z^{2}(A_{11}^{*}-\frac{A_{16}^{*2}}{A_{66}^{*}})ds+\frac{[{\oint {(A_{16}^{*}/A}_{66}^{*})zds]}^{2}}{\oint {(1/A}_{66}^{*})ds}+\oint D_{11}^{*}{\left(\frac{dy}{ds}\right)}^{2}ds,$$

(2e) $$C_{33}=\oint y^{2}(A_{11}^{*}-\frac{A_{16}^{*2}}{A_{66}^{*}})ds+\frac{[{\oint {(A_{16}^{*}/A}_{66}^{*})yds]}^{2}}{\oint {(1/A}_{66}^{*})ds}+\oint D_{11}^{*}{\left(\frac{dz}{ds}\right)}^{2}ds,$$

The coefficients were defined in a three-dimensional cartesian reference system (x,y,z), and the integral was computed along the curvilinear coordinate s, which followed the mid-thickness line of the generic closed section, which enclosed the area Ω. $A_{ij}^{*}$ and $D_{ij}^{*}$ were the coefficients of the reduced laminate extensional and bending laminate stiffness matrices. They were obtained from the coefficients $A_{ij}$ and $D_{ij}$ of the extensional and bending stiffness matrices [A] and [D], according to the classical laminate theory (CLT) and, in the case of symmetric lamination, with Equation (3), according to [28].

(3) $$A_{11}^{*}=A_{11}-\frac{A_{12}^{2}}{A_{22}},A_{16}^{*}=A_{16}-\frac{A_{12}A_{26}}{A_{22}},A_{66}^{*}=A_{66}-\frac{A_{26}^{2}}{A_{22}},\;D_{11}^{*}=D_{11}-\frac{D_{12}^{2}}{D_{22}},D_{16}^{*}=D_{16}-\frac{D_{12}D_{26}}{D_{22}},D_{66}^{*}=D_{66}-\frac{D_{26}^{2}}{D_{22}},$$

Considering the external loads $f_{v},f_{w}$, and $f_{\varphi }$, Equation (1) can be rewritten as:

(4) $$\left\{\begin{array}{c} \rho A\frac{\partial^{2}v}{\partial t^{2}}+C_{33}\frac{\partial^{2}}{\partial x^{2}}\left(\frac{\partial^{2}v}{\partial x^{2}}\right)=f_{v}\\ \rho A\frac{\partial^{2}w}{\partial t^{2}}+C_{33}\frac{\partial^{2}}{\partial x^{2}}\left(\frac{\partial^{2}w}{\partial x^{2}}\right)-C_{12}\frac{\partial }{\partial x}\left(\frac{\partial^{2}\varphi }{\partial x^{2}}\right)=f_{w}\\ \rho I_{p}\frac{\partial^{2}\varphi }{\partial t^{2}}-C_{11}\frac{\partial }{\partial x}\left(\frac{\partial \varphi }{\partial x}\right)+C_{12}\frac{\partial }{\partial x}\left(\frac{\partial^{2}w}{\partial x^{2}}\right)=f_{\varphi } \end{array}\right.,$$

Galerkin’s method was adopted to find the element stiffness and mass matrices. The method consists of expressing the variables v(x,t), $w\left(x,t\right)$, and φ(x,t) as a series of shape functions $\phi_{i}(x)$ multiplied for the degrees of freedom $\xi_{i}\left(t\right)$, which means:

(5) $$\left\{\begin{array}{c} v\left(x,t\right)=\sum_{j=i}^{N}\xi_{i}(t)\phi_{vi}(x)\\ w\left(x,t\right)=\sum_{j=i}^{N}\xi_{i}(t)\phi_{wi}(x)\\ \varphi \left(x,t\right)=\sum_{j=i}^{N}\xi_{i}(t)\phi_{\varphi i}(x) \end{array}\right.,$$

Once the shape functions were defined, the set of the function was multiplied by a residual function $R_{e}$ and integrated over the beam length, imposing zero as the solution for Equation (6)

(6) $$\int_{0}^{L}\phi_{i}R_{e}dx=0$$

In this way, the error between the shape function and the equation of the problem was minimized. The results of this procedure were the discrete equations of motion, written in the form:

(7) $$\left[M\right]\left\{\xi \right\}+\left[K\right]\left\{\xi \right\}=[F]$$

where $\left[M\right]$, $\left[K\right]$, and [F] represent the mass matrix, the stiffness matrix, and the vector of nodal forces, respectively.

Further details on shape functions, element stiffness, and mass matrices derivations can be found in [28].

The coefficients were constant along the element. For a beam structure with curvilinear stiffeners or fibers, the orientation $\vartheta_{i}$ at node 1 of the element was different from the orientation $\vartheta_{j}$ at node 2. In this case, the orientation of the stiffener along the element was considered equal to the mean value of ϑ at the nodes. This hypothesis was more accurate when the variation of ϑ inside the element was small. For stiffeners with high curvatures, this was achieved by increasing the number of elements for the beam structure. 

## 3. Problem Formulation

**Figure 2:**
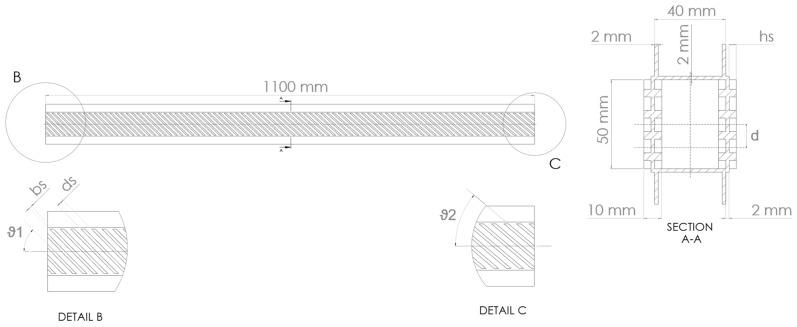
Beam structure geometry.

The generic box-beam structure analyzed in this work is depicted in Figure 2. It was characterized by a length of L=1100 mm, composed of two stiffened panels with a width of b=50 mm, and connected with two C-shaped spars with the dimension 20 mm×40 mm The stiffener dimensions were $h_{s}=4\;mm$ and $b_{s}=3\;mm$. The distance between the two stiffeners was $d_{s}=b/N=8.33\;mm$, where N=6 was the number of stiffeners. The stiffener orientations varied linearly with Equation (8), where $\vartheta_{1}$ was the orientation at the first section of the beam, while $\vartheta_{2}$ was the orientation at the end section. The thickness of the C-shaped spars and of the mid-layer of the stiffened panels was s=2 mm. The geometry followed the dimensions of the beam used in [28,29], where the structure was widely analyzed, and many numerical and experimental data are available for model verification; moreover, it can be manufactured and tested with the procedures and methods used in previous works. The material considered for the structural analysis was an Al6060 aluminum alloy, with its properties listed in Table 1.

The stiffener orientations evaluated at prescribed control points with Equation (8) were considered design variables in the topology optimization procedure: $\vartheta_{1}$ was the orientation at x=0, and $\vartheta_{2}$ was the orientation at x=L. The local orientations of the stiffeners were presumed to vary linearly, according to [41,42,43].

(8) $$\vartheta \left(x\right)=\vartheta_{1}+\frac{\left(\vartheta_{2}-\vartheta_{1}\right)}{b}x,$$

The optimization problem for the static bending–torsion coupling cases can be formulated as (9):

(9) $$\;max\;\frac{1}{2}{\left\{u\right\}}^{T}\left[K\right]\{u\},\;subject\;to\;\left[K\right]\left\{u\right\}=\{p\}\;\vartheta_{lb}\leq \vartheta_{1,2}\leq \vartheta_{ub}\;\left|\varphi_{tip}\right|\geq \left|\varphi_{0}\right|\;\left|w_{tip}\right|\leq \left|w_{0}\right|$$

While the optimization problem for the load cases where the objective is to find an uncoupling configuration can be written as (10):

(10) $$max\;\frac{1}{2}{\left\{u\right\}}^{T}\left[K\right]\{u\},\;subject\;to\;\left[K\right]\left\{u\right\}=\{p\}\;\vartheta_{lb}\leq \vartheta_{1,2}\leq \vartheta_{ub}\;\left.\varphi_{tip}\right.=0\;[{}^{\circ}]$$

where $\left[K\right]$ is the global stiffness matrix, $\left\{u\right\}$ is the vector of nodal displacements, and {p} is the vector of nodal moments and forces. In the case of planar deformation, for a finite element formulation, the strain energy was given by Equation (11). The static solution and the strain energy were obtained with a 100-element BTCE model constrained at one end.

(11) $$\frac{1}{2}{\left\{u\right\}}^{T}\left[K\right]\{u\},$$

The optimization was carried out with the MATLAB optimization algorithm “*fmincon*”. The allowable orientations ranged from $\vartheta_{lb}$ to $\vartheta_{ub}$, which represented the lower and upper boundaries of the problem. The stiffness coefficients $C_{11}$, $C_{12}$, $C_{22}$, and $C_{33}$ were computed with Equation (2); the CLT matrices were obtained by considering the stiffeners as equivalent orthotropic materials, according to [28,29,44], and with properties computed with Equation (12) and listed in Table 2. For each element, the stiffener orientations were considered equal to the mean angle between the angles at the element nodes.

(12) $$E_{11}=\left(\frac{E_{s}b_{s}}{d_{s}}\right);E_{22}=0;\nu_{12}=0;G_{12}=\frac{\tau_{y}^{s}}{4}\left(\frac{E_{s}b_{s}}{d_{s}}\right);G_{13}=0;G_{23}=0$$

The optimization was obtained with 200 initial couples of $\vartheta_{1}$ and $\vartheta_{2}$ randomly generated within the limit values of $\vartheta_{lb}$ and $\vartheta_{ub}$.

The optimization was performed by considering different load cases (LC) listed in Table 3 and different constraints on deformation. For each load case, the force F was equal to 41.37 kg, which was the same load considered in [27].

The first optimization was designed to find the configuration, which resulted in a coupled bending–torsion deformation with the highest level of strain energy and with constraints on maximum deflection and minimum torsion angle. In this case, the load was applied on the section centroid (LCi-C). The second optimization was arranged to find the configuration that separated bending and torsion when the load was applied at one-fourth of the beam width (LCi-U), which was equal to a distance of d=13 mm from the center of the beam section (see Figure 2). This parameter was chosen by assuming that the aerodynamic center of a hypothetical airfoil fixed to the box-beam structure was positioned at a distance d from the center of the closed section. These optimizations were referred to as “coupling optimization” and “uncoupling optimization”, respectively.

The constraints applied for each optimization are listed in Table 4, and those related to LC1_C, LC2_C, and LC3_C were the same ones used in [27].

The constraints reported in Table 4 for LC1_C, LC2_C, and LC3_C were the same constraints used in [27]. The design space for the case LC1_AM was modified in order to avoid or minimize the use of support structures during the AM process. For this reason, the minimum allowable angle was 40°. In general, it is not possible to determine “a priori” if the imposed constraints will be satisfied within the design space: the desired minimum torsion angle could be impossible to achieve with the imposed constraints on the deflection and vice-versa. Subsequent optimization cycles can be performed to refine the optimization, but for the scopes of this work, the configuration with the highest strain energy that was closer to the constraints was considered the best solution.

As an example of the application for beam structure design optimization, the optimal values related to $\vartheta_{1}$ and $\vartheta_{2}$ for each load case were rounded to the nearest angle with a precision of 0.5°, defining a set of design solutions with a slight variation in final deflections. Such a variation was considered acceptable for the scope of this work. A feasible design was so generated.

The chosen configurations were subsequently simulated with three different FE models: a 10 BTCE model, a SHELL FE model, and a TETRA10 FE model, following the procedure and the FE modeling described in [28]. For each model, the applied load was discretized with 10 concentrated loads positioned at element nodes for the BTCE and applied at the section centroid for the SHELL and the TETRA10 models. The three models were constrained at one end, imposing all the degrees of freedom equal to 0. The BTCE and the SHELL models consisted of ten sections. Each section considered a constant stiffener orientation equal to the mean value of the orientation angle at the section ends. The stiffened plates were considered laminates for the BTCE and the SHELL models, where the curvilinear stiffeners were modeled as an equivalent single layer. Their mechanical properties were computed by means of Equation (12).

**Figure 3:**
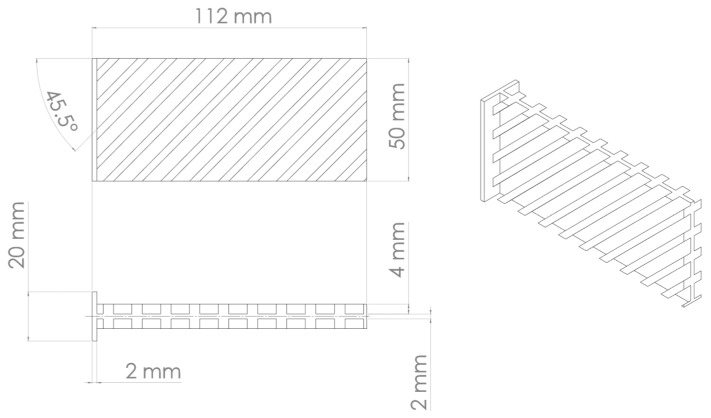
Stiffened panel for additive manufacturing simulation.

**Figure 4:**
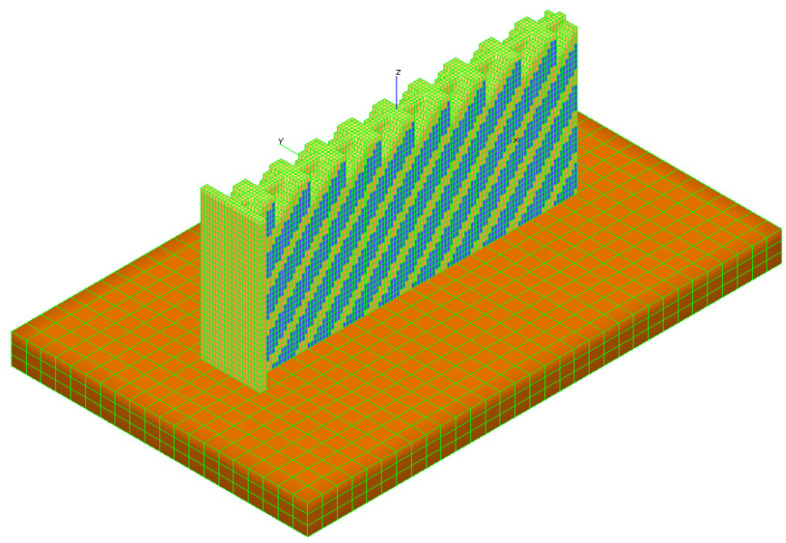
Finite element model for LPBF process simulation.

The additive manufacturing production of a portion of a stiffened panel, including related constraints, is represented in Figure 3 by means of a simulation performed with the AMTOP^®^ V. 2.0. AMTOP^®^ V. 2.0 is a platform of software tools developed to analyze and optimize additive manufacturing products and processes. The platform includes several algorithms to efficiently optimize the orientation of the part on the build platform [45] and evaluate the extent of stresses and distortions through a “layer-by-layer” approach [46,47]. The approach allows the macro-scale process simulation for most additive manufacturing technologies, such as powder melting (powder bed (PB) technology) or molten wire deposition (fused filament fabrication (FFF) technology), through a series of coupled thermal–structural finite element analyses. One of the approach assumptions is considering the size of the laser dot negligible, compared to the characteristic dimensions of the component. In the preprocessing step, an algorithm prepares the FE model starting from the surface mesh of the part with a technique called “voxelization” of the domain, i.e., discretization in layers on the growth plane, divided into small hexahedral subdomains. The voxels sizes are multiples of the layer thickness and the laser dot diameter. The software prepares the finite element model (Figure 4) of the part (in yellow), including powder elements (in blue) for stability purposes and the base plate elements (in orange); moreover, it sets the thermo-mechanical problem for the CalculiX solver, which evaluates the heat transfer in Equation (13) with boundary conditions reported in Equation (14). A representative flow of heat is applied to the entire layer for a time representative of the time of realization of the same layer. The layer realization time is the sum of the build time and the idle time for the repositioning of the re-coater (PB) or the nozzle (FFF).

Conduction, convection, and radiation heat transfer phenomena, as well as the non-linear mechanical elastic–plastic behavior, were taken into account in the analysis through the introduction of temperature-dependent material properties into the FE model. The evaluation of distortions of the part, compared to the nominal geometry, was carried out with the alignment of calculated and nominal part geometries through a root-mean-square error minimization method.

(13) $$\Delta \left(-\kappa \Delta T\right)+\rho c\dot{T}=\rho h$$

(14) $$-\kappa \Delta T=q-h_{c}\left(T-T_{0}\right)-\sigma_{e}\varepsilon (T^{4}-T_{0}^{4})$$

In Equations (13) and (14), T is the temperature, κ is the thermal conductivity of the material, ρ is the material density, h is the heat generation per unit of mass, q is the input heat flux, $h_{c}$ is the heat transfer coefficient under natural convection, $\sigma_{e}$ is the Stefan–Boltzmann constant, $T_{0}$ is the ambient temperature, and ε is the emissivity. The material considered for the simulation was a AlSi10Mg alloy, and the effects of emissivity and convection were neglected. The process parameters used for the simulation are listed in Table 5.

## 4. Results

**Figure 5:**
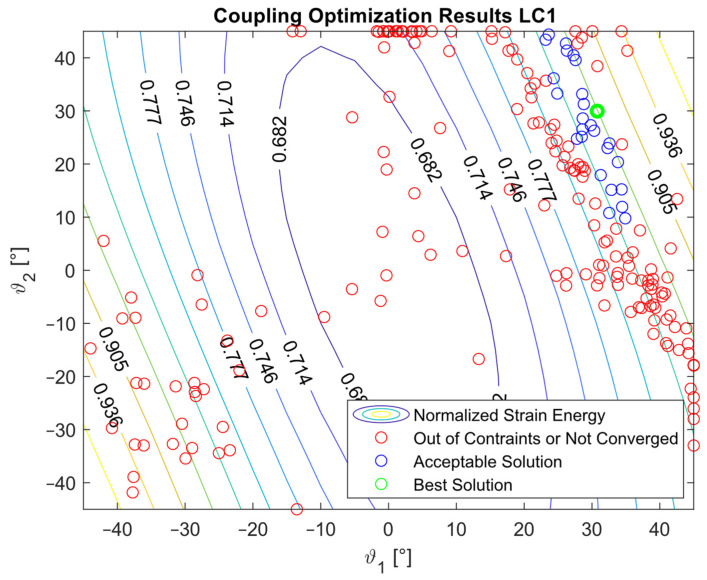
Optimization results for the LC1_C configuration.

**Figure 6:**
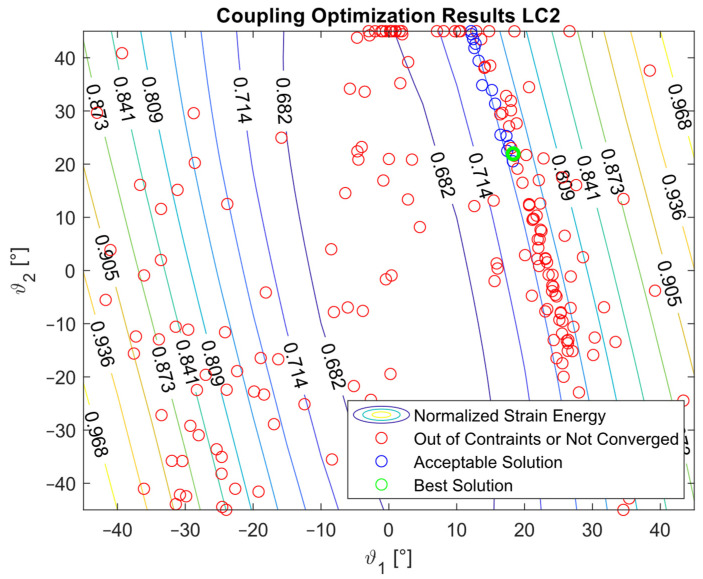
Optimization results for the LC2_C configuration.

**Figure 7:**
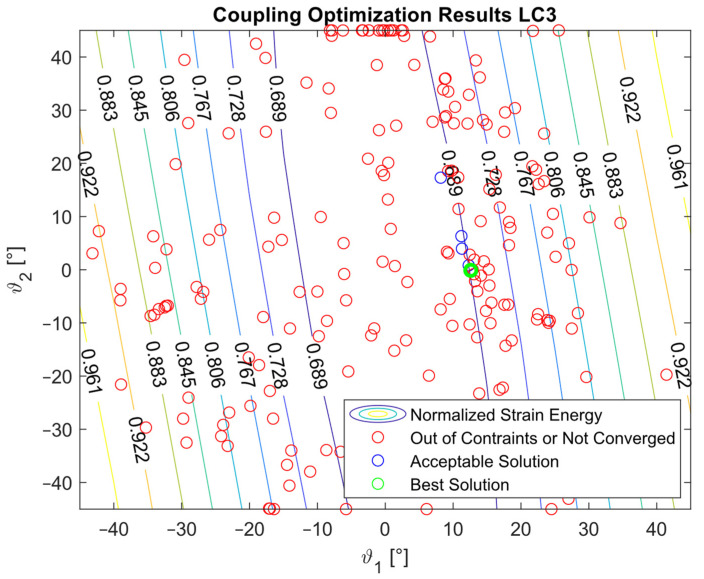
Optimization results for the LC3_C configuration.

**Figure 8:**
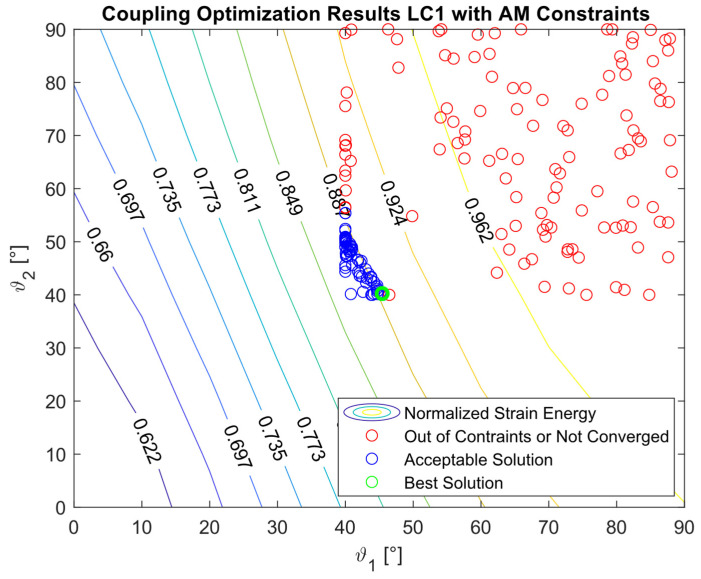
Optimization results for the LC1_AM configuration.

**Figure 9:**
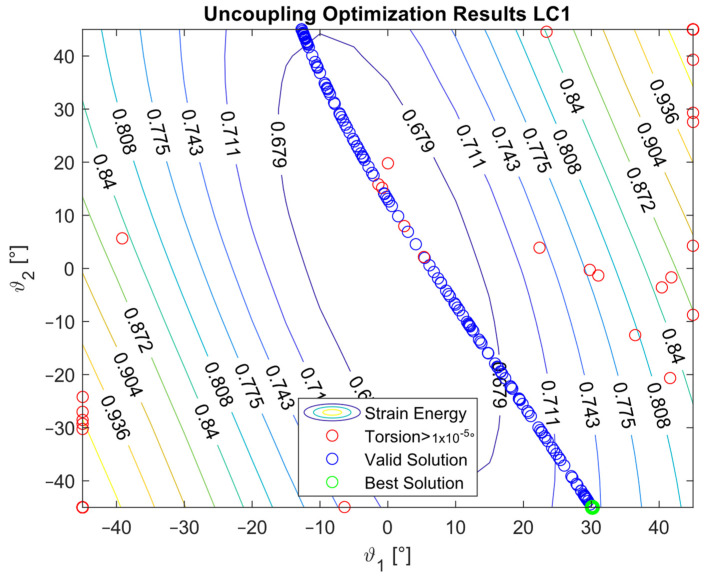
Optimization results for the LC1_U configuration.

**Figure 10:**
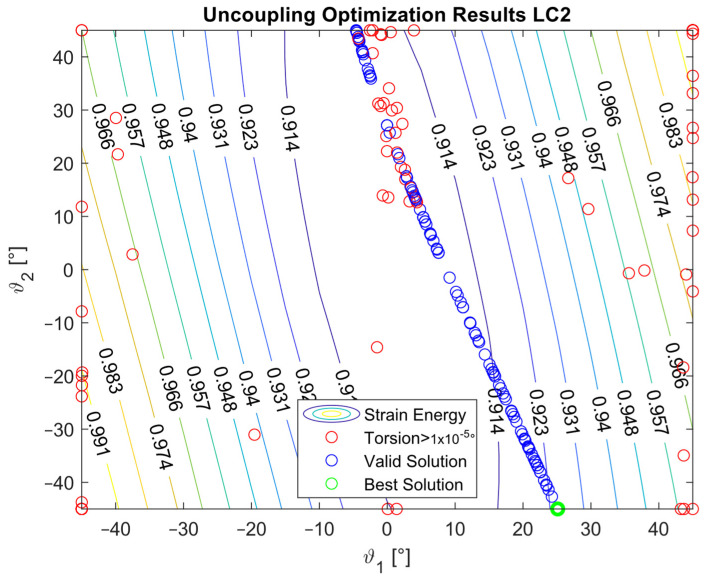
Optimization results for the LC2_U configuration.

**Figure 11:**
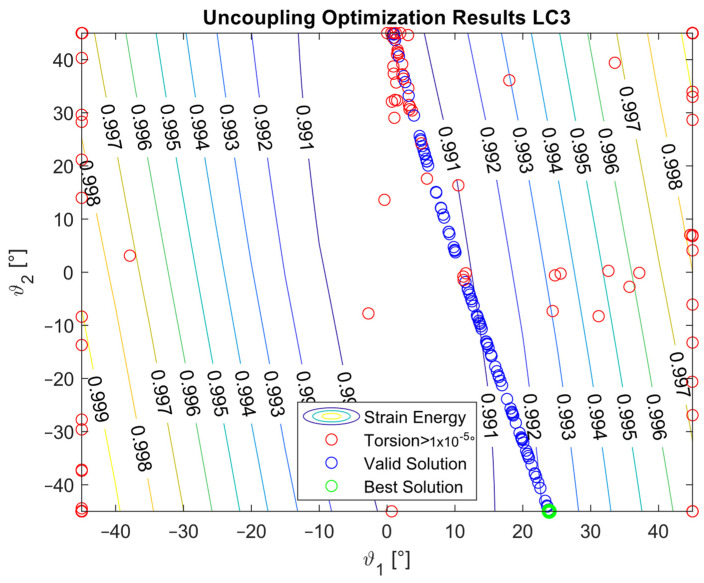
Optimization results for the LC3_U configuration.

The optimization procedure revealed that many different configurations gave a similar response and therefore a comparable strain energy level. However, it was possible to determine the configurations that achieved the highest strain energy values. The optimization results are presented in Figure 5, Figure 6, Figure 7, Figure 8, Figure 9, Figure 10 and Figure 11. The green dot represents the best solution, while the blue dots represent the solution with a tip deformation and a torsion angle with a 5% relative difference with respect to the best solution. These configurations were highlighted to show that the chosen optimization algorithm tended to converge to different solutions because there were multiple possible configurations that showed similar deformations and strain energy levels. The red dots represent the non-convergent solutions or configurations with tip deflections and torsion angles with a relative difference with respect to the best solution greater than 5%.

For the cases where the desired solution was meant to eliminate the bending–torsion couplings, it was interesting to note that the acceptable configurations laid on a line for each load case, and that the configurations with the highest strain energies were similar. The optimal solutions are listed in Table 6. Considering the introduced approximation related to FE discretization and the equivalent single layer representation, a rotation below 0.01° at the tip was considered equal to 0. It is worth noting that the best solution was placed at the boundary of the parameter range. In Figure 9, Figure 10 and Figure 11, all the other valid solutions were located on a curve, and consent to achieve the torsion angle was equal to zero; however, only the best solution had the highest strain energy level. This was plausible because the more the stiffeners approached an orientation of −90°, the more the bending stiffness decreased and the strain energy increased.

**Figure 12:**
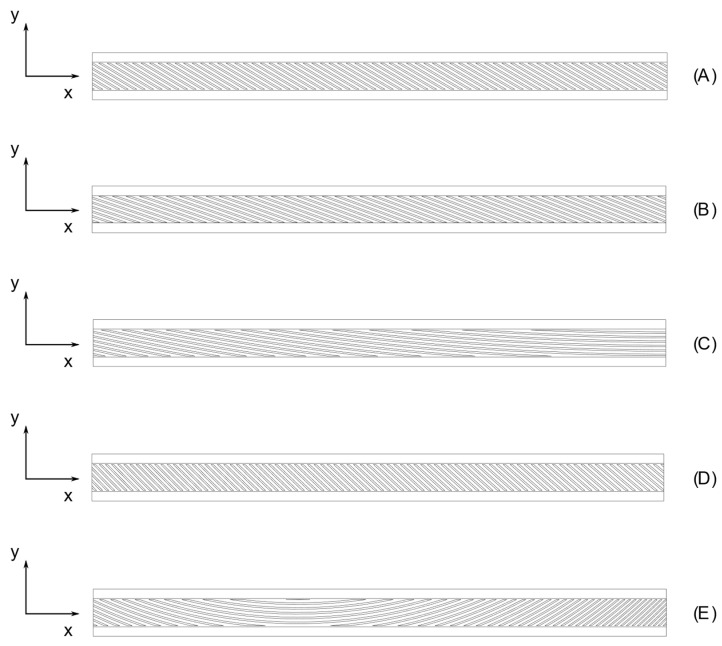
Configuration derived from optimal solutions: (**A**) LC1_C, (**B**) LC2_C, (**C**) LC3_C, (**D**) LC1_AM, and (**E**) LC1_U, LC2_U, and LC3_U.

The optimized angles for each configuration were approximated to the nearest value within 0.5°. Due to the similarities between the optimal solutions for the decoupling cases, a single configuration was chosen. The resulting configurations are listed in Table 7 and represented in Figure 12A–E.

Three different FE models were created for each one of the geometries presented in Table 7 and Figure 12A–E in order to compare the static results. The reference model was a TETRA10 FE model with a fully described geometry. Another model consisted of a SHELL FE model, with the beams divided in ten sections. For each section, the equivalent properties of the stiffeners were computed according to the procedure described in the previous section. The orientations of the stiffeners were considered uniform for each section and equal to the mean value of the angles at the two ends of the section. The third model consisted of a BTCE model with bending–torsion formulation; the finite element was the same one used for the optimization. Ten BTCE elements were considered for this model, and the orientations of the stiffeners were considered uniform along the beam element and computed with the same procedure used for the SHELL FE model.

**Figure 13:**
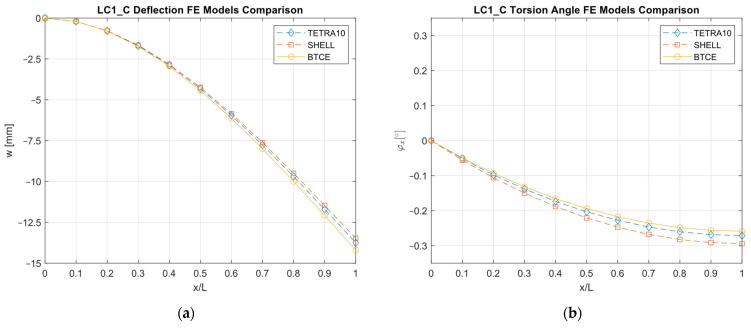
LC1_C deformation results comparisons between the TETRA10 FE model, SHELL FE model, and BTCE model: (**a**) deflection comparison, (**b**) torsion angle comparison.

**Figure 14:**
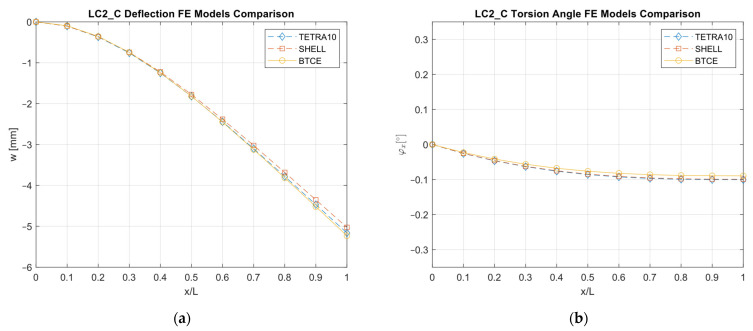
LC2_C deformation results comparisons between the vTETRA10 FE model, SHELL FE model, and BTCE model: (**a**) deflection comparison, (**b**) torsion angle comparison.

**Figure 15:**
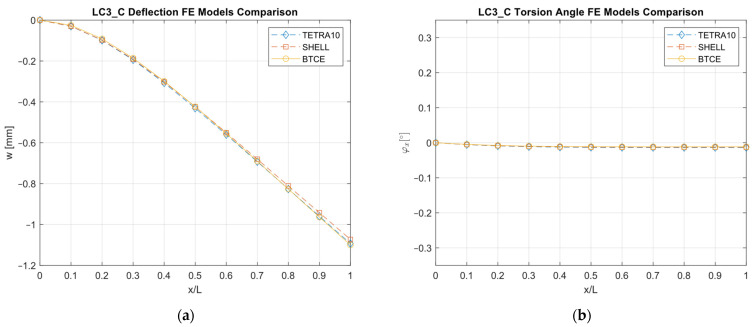
LC3_C deformation results comparisons between the TETRA10 FE model, SHELL FE model, and BTCE model: (**a**) deflection comparison, (**b**) torsion angle comparison.

**Figure 16:**
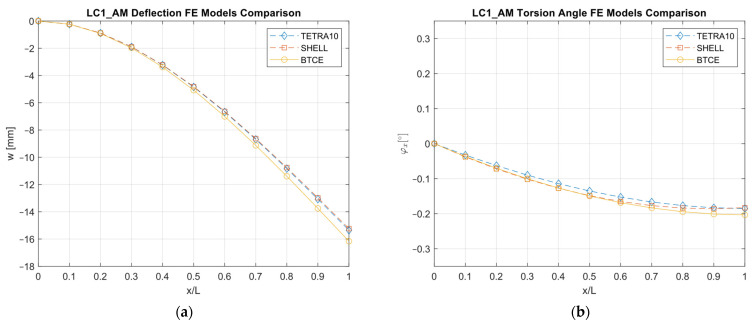
LC1_AM deformation results comparisons between the TETRA10 FE model, SHELL FE model, and BTCE model: (**a**) deflection comparison, (**b**) torsion angle comparison.

**Figure 17:**
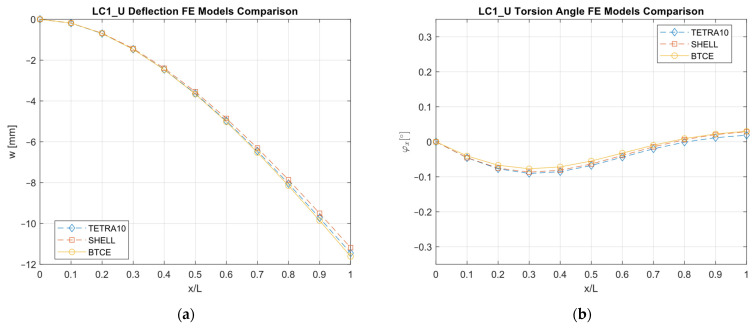
LC1_U deformation results comparisons between the TETRA10 FE model, SHELL FE model, and BTCE model: (**a**) deflection comparison, (**b**) torsion angle comparison.

**Figure 18:**
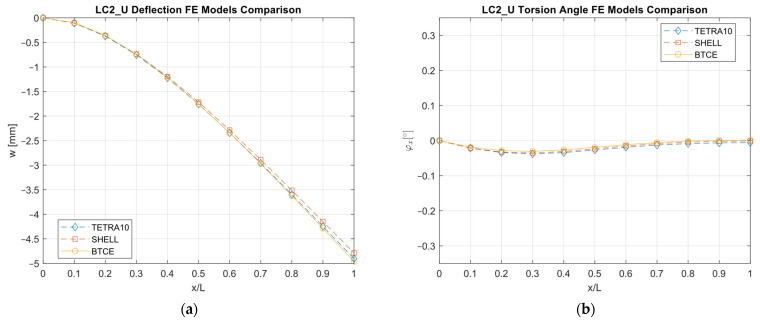
LC2_U deformation results comparisons between the TETRA10 FE model, SHELL FE model, and BTCE model: (**a**) deflection comparison, (**b**) torsion angle comparison.

**Figure 19:**
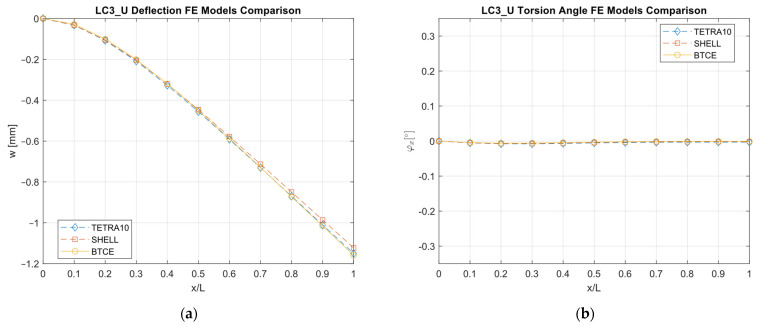
LC3_U deformation results comparisons between the TETRA10 FE model, SHELL FE model, and BTCE model: (**a**) deflection comparison, (**b**) torsion angle comparison.

The results obtained with the different FE models are reported in Figure 13, Figure 14, Figure 15, Figure 16, Figure 17, Figure 18 and Figure 19 and in Table 8.

In the majority of the considered cases, the geometries obtained from the optimal solution produced a deformation compliant with the design constraints. The static analysis revealed some differences with respect to the deformation results obtained with the optimal solutions reported in Table 5. These differences were linked to the approximations of $\vartheta_{1}$ and $\vartheta_{2}$, as introduced in the design procedure. In particular, the case LC2_C violated the imposed constraints on the deflection.

A good agreement between the different models were determined, showing a relative difference generally below 6% with respect to the TETRA10 FE model results and below 10% with respect to the SHELL FE model results. These differences were justified, considering the BTCE model assumptions. The developed beam element considered the stiffeners straight along the element length; moreover, an equivalent single layer material was adopted to describe the stiffened panel behaviors. In addition, the beam element section was considered non-deformable. However, the BTCE model demonstrated a good fidelity in representing the static behavior of a box-beam structure with curvilinear stiffener panels. This was a very interesting result for a tool useful in the preliminary design environment.

**Figure 20:**
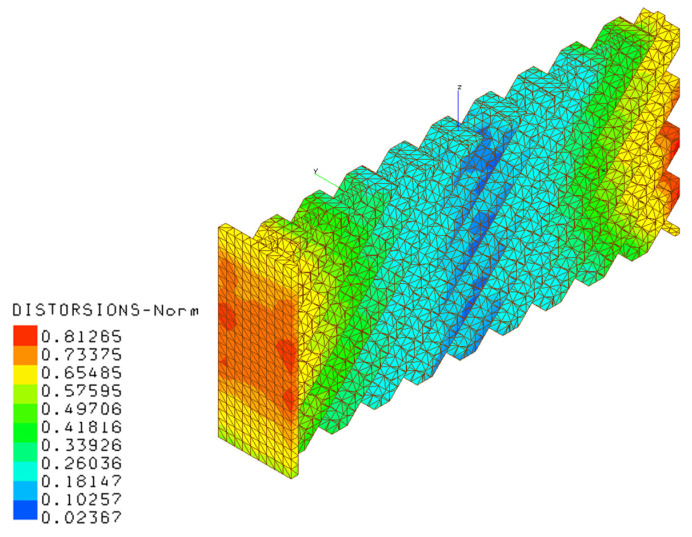
Deformation magnitudes for the AM component.

**Figure 21:**
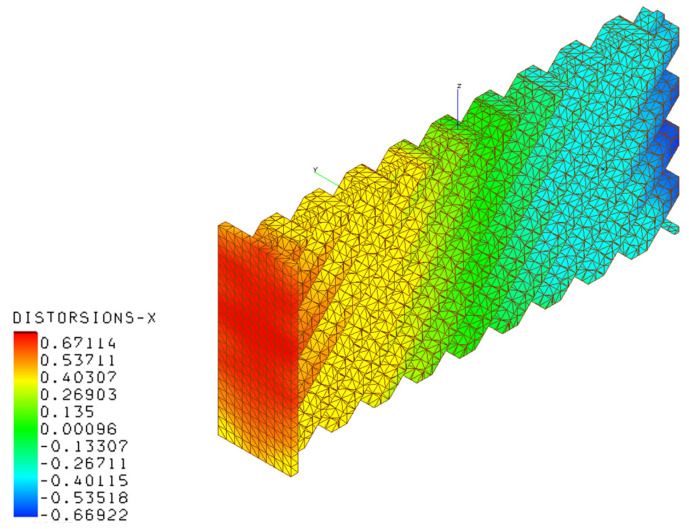
Deformations in the x-direction for the AM component.

**Figure 22:**
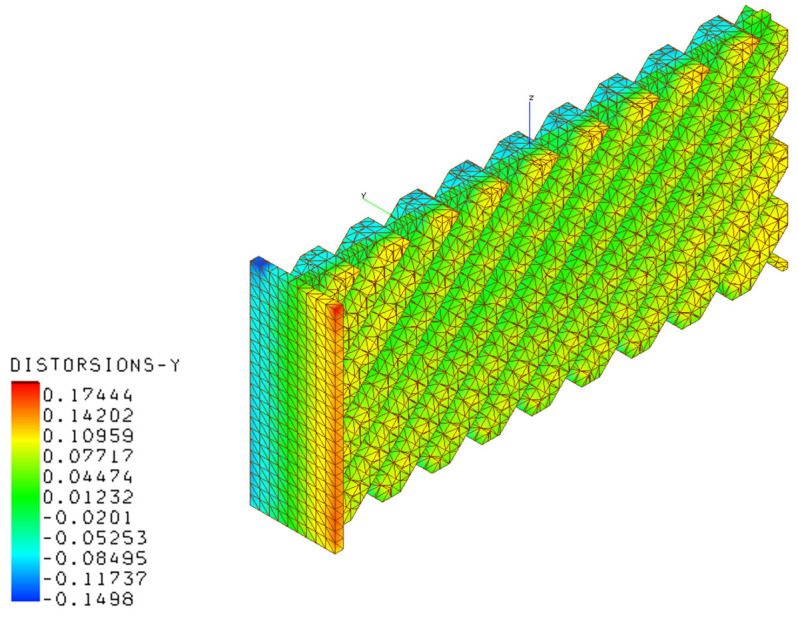
Deformations in the y-direction for the AM component.

**Figure 23:**
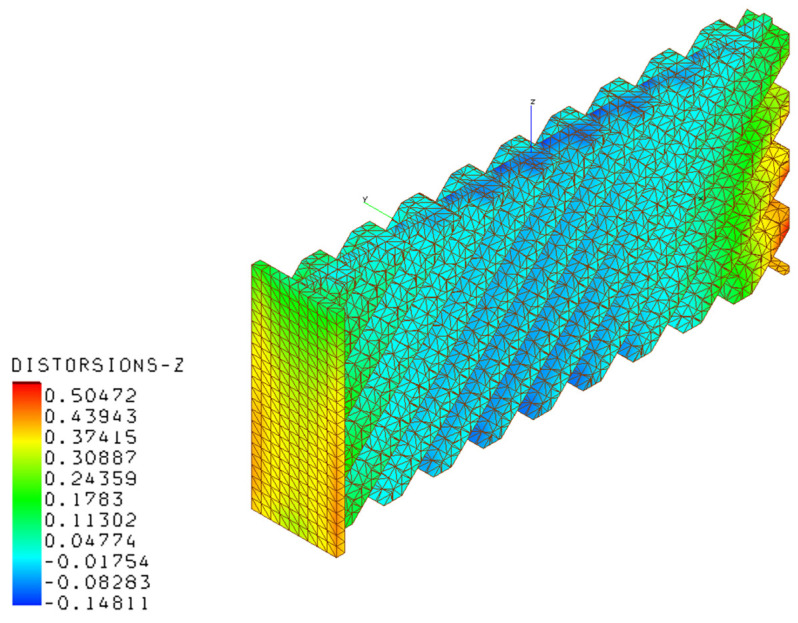
Deformations in the z-direction for the AM component.

Figure 20, Figure 21, Figure 22 and Figure 23 present the results of the thermo-mechanical simulations performed with AMTOP^®^; the reported results were displacements of the finite element model nodal coordinates from the starting geometry after the removal of the base plate and powder finite elements. The chosen process parameters and the designed geometry did not generate support structures minimizing the post-production machining. The deformations induced by the release of the stresses cumulated during the part cooling were represented in the principal directions. The simulation showed that the part could be produced with LPBF, obtaining a component with deformations lower than 0.82 mm.

## 5. Conclusions

The application of a beam finite element with bending–torsion formulation for the stiffener path optimization of box-beam structures with curvilinear stiffened panels was presented and validated. The optimization was devoted to three different load cases applied on the beam axis, including one typical situation related to manufacturing geometric constraints in the AM process. The objective function of the optimal procedure was focused on the maximum strain energy under the prescribed load related to the selected configuration under maximum vertical deflection and minimum torsional angle global constraints. With this method, the highest level of bending–torsion coupling was achieved.

A second optimization considered the same load cases but was positioned at one-fourth of the beam width, generating an additional torsional moment. In this case, the objective of the investigation was focused on minimizing the bending–torsion effect and therefore obtaining the maximum strain energy, with the torsion angle at the tip equal to 0.

The optimization results were converted into a beam structure design to assess the structural performances of the chosen configurations and to verify whether the BTCE was capable of correctly representing the deformation under static loads of beam structures with curvilinear stiffeners. A TETRA10 FE model, a SHELL FE model, and a BTCE model were created for each design, and a static analysis was performed. The comparison between the different FE models revealed a good precision of the BTCE, with relative differences in the deformation results less than 6% in most of the cases when compared to the TETRA10 FE model results and less than 10% when compared to the SHELL FE model. The discrepancies between the different models can be explained by considering the assumptions made for the BTCE derivation. The developed beam element considered the stiffeners straight along the element length and reduced the stiffeners to an equivalent single layer. In addition, the beam element section was considered non-deformable.

The potential of the BTCE as an optimization tool and as a static analysis tool for beam structures with curvilinear stiffeners was confirmed. Moreover, the possibility to obtain a specific configuration capable of enhancing or diminishing the effect of bending on torsion and vice-versa was also demonstrated.

The optimal configuration related to geometric constraints for AM production with a self-supporting structure that minimized the post-production machining was also performed. AMTOP^®^ thermo-mechanical simulation revealed that residual stress induced deformations smaller than 0.82 mm on the final component, confirming the possibility for the AM production of stiffened panels for such performant beam structures.

## Figures and Tables

**Table 1:** Al6060 mechanical properties.

Property	Value
Young’s Modulus, E [MPa]	58,000
Shear Modulus, G [MPa]	21,805
Poisson’s ratio, $\nu_{12}$	0.33

**Table 2:** Equivalent single-layer material properties.

Property	Value
Longitudinal Young’s Modulus, $E_{11}$ [MPa]	20,888.36
Transverse Young’s Modulus, $E_{22}$ [MPa]	0
Shear Modulus, $G_{12}$ [MPa]	1636.03
Poisson’s ratio, $\nu_{12}$	0

**Table 3:** Load cases descriptions.

Load Case	Equation	Graphical Representation
LC1	$q_{w}=F$	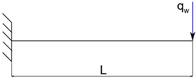
LC2	$q_{w}=\frac{F}{L}$	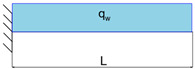
LC3	$q_{w}=\frac{F}{L}\left(1-\frac{x}{L}\right)$	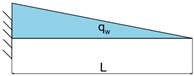

**Table 4:** Optimizations loads, design limits, and constraints.

Optimization	Load Applied	${[\vartheta }_{lb}\;\vartheta_{ub}]$	$Constraints\;{[\varphi }_{0}\;w_{0}]$
LC1_C	LC1	[−45° 45°]	[0.287° 14 mm]
LC2_C	LC2	[−45° 45°]	[0.08° 4.6 mm]
LC3_C	LC3	[−45° 45°]	[0.03° 1.1 mm]
LC1_AM	LC1	[40° 90°]	[0.2° 17 mm]
LC1_U	LC1 d=13mm	[−45° 45°]	$\varphi_{tip}$ < 1 × 10^−5^ [rad]
LC2_U	LC2 d=13mm	[−45° 45°]	$\varphi_{tip}$ < 1 × 10^−5^ [rad]
LC3_U	LC3 d=13mm	[−45° 45°]	$\varphi_{tip}$ < 1 × 10^−5^ [rad]

**Table 5:** AM process simulation process parameters.

Process Parameters	Value
Recoat Time [s]	10
Layer Thickness (LT) [mm]	0.03
Hatch Distance [mm]	0.2
Base Plate Temperature [°C]	160
Laser Diameter (LD) [mm]	0.1
Laser Speed [mm/s]	1200
Element Width	10× LD
Element Height	50× LT

**Table 6:** Optimal solutions.

Optimization	Applied Load	${[\vartheta }_{1}\;\vartheta_{2}]$	$w_{tip}\;[mm]$	$\varphi_{tip}$ **[°]**
LC1_C	LC1	[30.77° 29.96°]	−13.98	−0.26
LC2_C	LC2	[18.32° 21.95°]	−4.60	−0.08
LC3_C	LC3	[12.50° −0.11°]	−1.10	−0.01
LC1_AM	LC1	[45.43° 40.24°]	−15.93	−0.2
LC1_U	LC1 d=13 mm	[30.16° −45°]	−11.88	$\left|\varphi_{tip}\right|$ < 0.01
LC2_U	LC2 d=13 mm	[25.10° −45°]	−4.39	$\left|\varphi_{tip}\right|$ < 0.01
LC3_U	LC3 d=13 mm	[23.88° −45°]	−1.15	$\left|\varphi_{tip}\right|$ < 0.01

**Table 7:** Configurations derived from optimal solutions.

Optimization	Applied Load	${[\vartheta }_{1}\;\vartheta_{2}]$
LC1_C	LC1	[31° 30°]
LC2_C	LC2	[18.5° 22°]
LC3_C	LC3	[12.5° 0°]
LC1_AM	LC1	[45.5° 40°]
LC1_U	LC1 d=13 mm	[25° −45°]
LC2_U	LC2 d=13 mm	[25° −45°]
LC3_U	LC3 d=13 mm	[25° −45°]

**Table 8:** Finite elements model Results Comparisons.

**Coupled Bending–Torsion**				
**Load Case**	**DOF**	**BTCE**	**SHELL**	**TETRA10**
LC1	*w* [mm]	−14.20	−13.47	−13.75
			(5.4%)	(3.3%)
	$\varphi_{x}$ [°]	−0.26	−0.29	−0.27
			(10.3%)	(3.7%)
LC2	*w* [mm]	−5.23	−5.03	−5.16
			(4.0%)	(1.4%)
	$\varphi_{x}$ [°]	−0.09	−0.10	−0.10
			(10%)	(10%)
LC3	*w* [mm]	−1.10	−1.07	−1.09
			(0.9%)	(0.9%)
	$\varphi_{x}$ [°]	−0.01	−0.01	−0.01
			(0%)	(0%)
LC1 AM	*w* [mm]	−16.17	−15.24	−15.34
			(6.1%)	(5.4%)
	$\varphi_{x}$ [°]	−0.20	−0.18	−0.19
			(11.1%)	(5.26%)
**Uncoupled Bending–Torsion**				
**Load Case**	**DOF**	**BTCE**	**SHELL**	**TETRA10**
LC1	*w* [mm]	−11.61	−11.19	−11.45
			(3.8%)	(1.4%)
	$\varphi_{x}$ [°]	0.03	0.03	0.02
			(0%)	(50%)
LC2	*w* [mm]	−4.96	−4.79	−4.90
			(3.5%)	(1.2%)
	$\varphi_{x}$ [°]	$-0.01\;<\;\varphi_{x}$ < 0.01	$-0.01\;<\;\varphi_{x}$ < 0.01	$-0.01\;<\;\varphi_{x}$ < 0.01
LC3	*w* [mm]	−1.16	−1.12	−1.15
			(3.6%)	(0.9%)
	$\varphi_{x}$ [°]	$-0.01\;<\;\varphi_{x}$ < 0.01	$-0.01\;<\;\varphi_{x}$ < 0.01	$-0.01\;<\;\varphi_{x}$ < 0.01

## Data Availability

Data available upon request.
